# Direct Oral Anticoagulants: From Randomized Clinical Trials to Real-World Clinical Practice

**DOI:** 10.3389/fphar.2021.684638

**Published:** 2021-05-26

**Authors:** Roberta Roberti, Luigi Francesco Iannone, Caterina Palleria, Antonio Curcio, Marco Rossi, Angela Sciacqua, Giuseppe Armentaro, Ada Vero, Antonia Manti, Velia Cassano, Emilio Russo, Giovambattista De Sarro, Rita Citraro

**Affiliations:** ^1^ Science of Health Department, School of Medicine, University “Magna Graecia” of Catanzaro, Catanzaro, Italy; ^2^ Department of Medical and Surgical Sciences, University “Magna Graecia” of Catanzaro, Catanzaro, Italy; ^3^ Department of Experimental and Clinical Medicine, University “Magna Graecia” of Catanzaro, Catanzaro, Italy

**Keywords:** Direct oral anticoagulants, pharmacology, safety, drug–drug interactions, randomized clinical trials, clinical practice

## Abstract

Direct oral anticoagulants (DOACs) are a more manageable alternative than vitamin K antagonists (VKAs) to prevent stroke in patients with nonvalvular atrial fibrillation and to prevent and treat venous thromboembolism. Despite their widespread use in clinical practice, there are still some unresolved issues on optimizing their use in particular clinical settings. Herein, we reviewed the current clinical evidence on uses of DOACs from pharmacology and clinical indications to safety and practical issues such as drugs and food interactions. Dabigatran is the DOAC most affected by interactions with drugs and food, although all DOACs demonstrate a favorable pharmacokinetic profile. Management issues associated with perioperative procedures, bleeding treatment, and special populations (pregnancy, renal and hepatic impairment, elderly, and oncologic patients) have been discussed. Literature evidence shows that DOACs are at least as effective as VKAs, with a favorable safety profile; data are particularly encouraging in using low doses of edoxaban in elderly patients, and edoxaban and rivaroxaban in the treatment of venous thromboembolism in oncologic patients. In the next year, DOAC clinical indications are likely to be further extended.

## Introduction

The introduction of direct oral anticoagulants (DOACs) has induced a thorough frameshift in the primary and secondary prevention of ischemic stroke in patients with nonvalvular atrial fibrillation (NVAF) as well as in prevention and treatment of venous thromboembolism (VTE), namely, pulmonary embolism and deep venous thrombosis (Chan et al., 2020). Indeed, despite the anticoagulant treatment with vitamin K antagonists (VKAs) has been the standard therapy for more than 60 years (Joppa et al., 2018), due to the rapid increase in robust evidence provided by randomized clinical trials (RCTs) and greater manageability, the DOACs are now widely used in clinical practice worldwide.

Two classes of DOACs are currently available: reversible direct thrombin inhibitors (i.e., dabigatran) and the direct inhibitors of Xa factor (i.e., rivaroxaban, apixaban, and edoxaban). Compared to VKAs, these target-specific drugs have a fixed dosing with no need for international normalized ratio (INR) monitoring, a wider therapeutic index, rapid onset and short half-lives, and few drug and food interactions (Sikorska and Uprichard, 2017; Weitz et al., 2017). In addition to non-inferior efficacy, DOACs have also shown a better safety profile in RCTs than VKAs (Weitz et al., 2017; Steffel et al., 2018). However, some unresolved questions on the optimal use of these drugs in specific clinical situations (e.g., patients with malignancy or other comorbidities and frail patients) remain.

Herein, we provide an overview of DOACs’ main characteristics and advantages focusing on safety and practical issues, such as drug and food interactions, bridging in perioperative procedures, bleeding treatment, and special populations management (i.e., pregnancy, renal and hepatic impairment, elderly, and oncologic patients).

## Clinical Indications and Pharmacology

Nowadays, DOACs have been approved by regulatory authorities for use in specific conditions including prevention of stroke and systemic embolism in nonvalvular AF with one or more risk factors (i.e., prior stroke, transient ischemic attack or systemic embolism, age ≥75 years, hypertension, diabetes mellitus, heart failure, or left ventricular ejection ≤30%) and for primary and secondary treatment of VTE (Joppa et al., 2018). Rivaroxaban has also been approved for prevention of cardiovascular events after acute coronary syndrome (Mega et al., 2012; Plosker, 2014).

The DOACs act as anticoagulants through the direct inhibition of two different and specific factors on the coagulation pathway, in contrast to the indirect, posttranslational, and wide inhibition by VKAs. Apixaban, rivaroxaban, and edoxaban reversibly inhibit Xa factor, regardless of status (free or in a thrombus). Dabigatran acts downstream of the coagulation pathway, inhibiting reversibly and selectively IIa factor (thrombin), both in thrombus and free (Wang and Bajorek, 2014). Pharmacokinetic and safety profiles differ substantially among DOACs. A patient-tailored therapy must consider these differences in addition to overall clinical conditions (e.g., comorbidities and other treatments). The main pharmacokinetic characteristics are summarized in Table 1.

**TABLE 1:** Summary of main pharmacokinetic characteristics of DOACs.

Characteristic	Dabigatran	Rivaroxaban	Apixaban	Edoxaban
Prodrug	Yes	No	No	No
Bioavailability (%)	3–7 (due to its high polarity)	70 (without food) 100 (with food)	50	62
Time to maximum effect [T_max_ (h)]	1.5–2 h	2–4 h	1–3 h	1–2 h
Volume of distribution [VD (L)]	50–70	50	23	107
Plasma protein binding (%)	35	>90	87	55
Half-life (h)	12–14	5–9 (young adults) 11–13 (elderly)	∼12	10–14
Metabolism	No (20% glucuronic acid conjugation)	(65%) CYP3A4, CYP2J2	(73%) CYP3A4/5, 1A2, 2C8, 2C9, 2C19, 2J2	(50%) CYP3A4/5 (<10%)
Substrate for CYP3A4	No	Yes	Yes	Yes
Substrate for P-gp	Yes, dabigatran etexilate	Yes	Yes	Yes
Substrate for other transporters	Unknown	BCRP	BCRP	Unknown
Elimination	80% renal (unchanged) 20% liver	33% renal 66% liver	25% renal 75% liver	50% renal 50% liver
Drug–drug interactions	P-gp	P-gp, CYP3A4	P-gp, CYP3A4	P-gp, CYP3A4
Food–drug interactions	Prolongs T_max_ to 2 h (Intake with food discouraged)	Mean AUC increases to ≈40% (Intake with food mandatory)	No effect (Intake with food discouraged)	No effect (Intake with food: no official recommendation)
Daily doses required	Twice daily	Once daily	Twice daily	Once daily

BCRP: breast cancer–resistant gene protein, CYP: cytochromes P450, P-gp: P-glycoprotein.

### Summary of Direct Oral Anticoagulants Studies in Cardiovascular Disorders

Nowadays, several non-inferiority, randomized, double-blind, and international trials have been performed to evaluate DOACs’ efficacy in preventing stroke and systemic embolic events in patients with nonvalvular AF compared with warfarin and have been largely described in the literature (Table 2) (Connolly et al., 2009; Granger et al., 2011; Patel et al., 2011; Giugliano et al., 2013).

**TABLE 2:** DOAC safety and efficacy compared to Warfarin.

	Efficacy outcome		Safety outcomes	
	Stroke or systemic embolism (SE) (%/year)	Major bleeding (%/year)	Intracranial bleeding (%/year)	Gastrointestinal bleeding (%/year)
Dabigatran 110 mg^a^	1.53	2.71	0.23	1.12
Dabigatran 150 mg	1.11	3.11	0.30	1.51
Warfarin	1.69	3.36	0.74	1.02
Rivaroxaban 20 mg^b^	1.7	3.6	0.5	3.2
Warfarin	2.2	3.4	0.7	2.2
Apixaban 5 mg^c^	1.27	2.13	0.33	0.76
Warfarin	1.60	3.09	0.80	0.86
Edoxaban 30 mg^d^	Stroke 1.91	1.61	0.26	0.82
	SE 0.15			
Edoxaban 60 mg	Stroke 1.49	2.75	0.39	1.51
	SE 0.08			
Warfarin	Stroke 1.69	3.43	0.85	1.23
	SE 0.12			

a Connolly et al. (2009).

b Patel et al. (2011).

c Granger et al. (2011).

d Giugliano et al. (2013).

Pooled efficacy and safety data of DOACs used for on-label cardiovascular indications and considered as a single drug class showed that compared with VKAs, DOACs have a favorable efficacy profile in patients with nonvalvular AF (lower risk to develop stroke or systemic embolism, pooled OR 0.76) and comparable efficacy in patients with VTE. The overall safety profile resulted favorable, with an all-cause mortality lower or comparable with VKAs and lower bleeding risk (except for gastrointestinal bleedings, which were higher in patients with nonvalvular AF taking rivaroxaban or dabigatran) (Makam et al., 2018).

Oral anticoagulant therapy has also been evaluated in the secondary prevention of coronary artery disease (CAD). In patients with CAD, acute coronary syndrome (ACS), percutaneous coronary intervention (PCI), and coexisting AF, bleeding risk is increased by combined antiplatelet therapy. Several studies have been conducted to assess DOACs’ efficacy and safety in this field and have been recently described in a review which evaluated the current standard of care in secondary prevention of CAD (stable CAD and ACS) with antithrombotic agents in patients both with and without AF, with invasive or conservative management. Bearing in mind that a highly personalized approach is required for all these patients and those with AF, evidence suggests the use of DOACs, rather than VKAs, to reduce bleeding risk; low-dose oral anticoagulation might represent a future therapeutic strategy in stable CAD or after dual antiplatelet therapy discontinuation (Verheugt et al., 2019).

Moreover, although DOACs are contraindicated in patients with mechanical heart valves, limited evidence from subgroup analyses of RCTs and cohort studies seems not to preclude their use in AF patients with bioprosthetic valves (Steffel et al., 2018). Finally, DOACs’ efficacy and safety have been investigated in peripheral arterial disease (PAD). In the multinational, multicenter, double-blind VOYAGER-PAD trial, 6,564 patients were randomly assigned in a 1:1 ratio to receive low-dose rivaroxaban (2.5 mg BID) plus aspirin or placebo plus aspirin. All patients had documented lower extremity PAD and underwent revascularization within the previous 10 days. The primary efficacy outcome was a composite of acute limb ischemia, amputation for vascular causes, myocardial infarction, ischemic stroke, or cardiovascular death; major bleeding as defined by the Thrombolysis in Myocardial Infarction (TIMI) was assessed as the main safety outcome. Patients treated with low-dose rivaroxaban plus aspirin showed an approximately 15% reduction in the composite outcome incidence, with no significant differences in the safety outcome among the two groups. However, with the International Society on Thrombosis and Haemostasis (ISTH) definition of major bleeding, it resulted higher in the rivaroxaban plus aspirin group (Bonaca et al., 2020).

### Pharmacokinetics and Drug–Drug Interactions

#### Dabigatran

Dabigatran is administered as a prodrug (dabigatran etexilate) due to its high polarity that precludes gastrointestinal absorption. Indeed, compared to dabigatran, the etexilate form is less basic and hydrophilic to enhance absorption (dependent on intestinal permeability glycoprotein [P-gp] transport) and drug dissolution (Stangier et al., 2005). It is rapidly hydrolyzed to active dabigatran by plasma esterase (Stangier et al., 2008; Fawzy and Lip, 2019). Food intake can significantly delay the drug absorption time (Wessler et al., 2013) but does not modify the bioavailability (usually 3–7%) (Stangier et al., 2008). After absorption, the drug has a moderate volume of distribution (V_d_) ranging from 50 to 70 L and a poor plasma protein binding (35%), with a small percentage (20%) metabolized by conjugation reactions (see Table 1 for more details) (Blech et al., 2008; Fawzy and Lip, 2019). No oxidative reactions have been identified so far. Dabigatran is mainly excreted unmodified by kidneys, and dose adjustments in patients with renal impairment are required, whereas it is contraindicated in the severe renal impairment to prevent drug accumulation and the subsequent increase risk of major bleeding (Bellamy et al., 2009; Becattini et al., 2018).

#### Rivaroxaban

Rivaroxaban is rapidly absorbed and reaches peak plasma concentration in 2–4 h (Kreutz, 2014). Food intake enhances bioavailability of 15 and 20 mg doses, probably increasing the drug solubilization and dissolution (Stampfuss et al., 2013; Fawzy and Lip, 2019). Coadministration with food is associated with a reduced interindividual variability, increasing rivaroxaban plasma concentration predictability (Kubitza et al., 2006). Rivaroxaban is a substrate for both P-gp transporters and breast cancer–resistant gene protein (BCRP) (Gnoth et al., 2011), has a moderate V_d_ (50 L), and is highly bound (>90%) to plasma proteins. Predominantly metabolized in the liver, mainly by cytochrome P450 (CYP), enzymes 3A4 and 2J2, and other CYP-independent pathways (Weinz et al., 2009), rivaroxaban is eliminated through renal and hepatobiliary routes with almost a third of the drug excreted unmodified in the urine (Weinz et al., 2009). Dose adjustments are required in patients with renal and hepatic impairment (De Caterina et al., 2012).

#### Apixaban

Apixaban is primarily absorbed in the small intestine, and different from rivaroxaban and dabigatran, the presence of food does not affect bioavailability (which is approximately of 50%) (Frost et al., 2013a; Frost et al., 2013b). It is a substrate of P-gp and BCRP transporters (De Caterina et al., 2012), with a high binding rate to plasma proteins (87%) (Fawzy and Lip, 2019) and the lowest V_d_ (approximately 23 L) compared to other DOACs (Wong et al., 2011). Apixaban is primarily metabolized by CYP3A4/3A5 and secondarily by sulfotransferase (SULT) 1A1 (Raghavan et al., 2009) with drug elimination by renal (25%) and hepatobiliary (75%) routes.

Dose adjustments are not usually required. Particular caution in patients with severe renal impairment is mandatory and should not be used with a glomerular fraction rate (GFR) < 15 ml/min (Chang et al., 2016). Apixaban may also be used with particular caution in patients with mild to moderate hepatic impairment and contraindicated in severe liver disease (De Caterina et al., 2012).

#### Edoxaban

Edoxaban is mainly absorbed in the small intestine, with an absolute oral bioavailability of 62%, regardless of food intake (Bounameaux and Camm, 2014), depending on P-gp transporter as other DOACs. The drug is bound to plasma proteins for almost 55% and extensively distributed throughout the body with a large V_d_ (107L) (Fawzy and Lip, 2019). The drug is slightly metabolized by cytosol carboxyl esterase-1 enzyme, liver microsomes, CYP3A4 enzymes, and glucuronidation reactions (Table 1) (Parasrampuria and Truitt, 2016; Gelosa et al., 2018). Edoxaban is eliminated unchanged in urine (50%) and through biliary secretion (50%) (Plitt and Giugliano, 2014). Renal impairment increases limitedly the drug plasma concentrations, with no significant difference between moderate and severe renal failure (Parasrampuria and Truitt, 2016); dosage modifications are not recommended. Likewise, mild to moderate hepatic impairment minimally affects edoxaban pharmacokinetic, but the drug is not indicated in severe hepatic impairment (Steffel et al., 2018).

#### Drug–Drug Interactions

Bearing in mind the role of the P-gp transporter in DOACs absorption, coadministration with strong P-gp inducers or inhibitors is contraindicated. Indeed, P-gp inhibitors, including several drugs, largely used in patients with AF (i.e., verapamil, dronedarone, or amiodarone) but also anti-mycotic medications, antibiotics belonging to macrolides, and antiretroviral protease inhibitors could increase DOACs plasma concentrations with an increased risk of bleeding.

In the multivariate analysis of a noninterventional prospective study on elderly patients (>85 years), bleeding risk increased about six-fold with dabigatran and a P-gp inhibitor (e.g., amiodarone) coadministration (Bernier et al., 2019). On the other hand, strong P-gp inducers such as rifampicin or hypericum could reduce DOAC concentrations to subclinical dose (Gong and Kim, 2013). However, edoxaban can be coadministered with quinidine, verapamil, and amiodarone at the dose of 60 mg/day and with cyclosporine, dronedarone, erythromycin, and ketoconazole, halving the dose (Corsini et al., 2020).

In addition to P-gp, apixaban and rivaroxaban are also substrates of BRCP transporters, but the clinical significance of this transporter has not been investigated yet (Fawzy and Lip, 2019). No drug–drug interactions (DDIs) involving CYP450 enzymes with dabigatran have been reported, whereas concomitant administration of rivaroxaban, apixaban, and edoxaban with CYP3A4 strong inhibitors or inducers is not recommended (De Caterina et al., 2012; Steffel et al., 2018). In a retrospective cohort study, coadministration of P-gp and moderate CYP3A4 inhibitors (i.e., amiodarone, dronedarone, diltiazem, verapamil, or erythromycin) with rivaroxaban or apixaban for at least 3 months has been associated with a higher overall bleeding risk than rivaroxaban or apixaban alone (*p* = 0.006) (Hanigan et al., 2020).

Edoxaban clearance is increased by 33% with a half-life reduction of 50% in coadministration with rifampicin, a strong P-gp inducer. However, no significant changes in PT and aPTT have been described in clinical practice, and the concurrent use is not contraindicated but requires particular care (Mendell et al., 2015).

Histamine H2 receptor antagonists or proton pump inhibitors, through the increase in pH levels, could reduce dabigatran absorption (12 and 30%, respectively), but dose modifications are not required (Heidbuchel et al., 2013). Finally, all DOACs are not indicated in patients treated with antiretroviral protease inhibitors, tyrosine kinase inhibitors, dexamethasone, levetiracetam, and valproic acid (Steffel et al., 2018).

In a case–control study, pharmacokinetic interactions do not seem to increase the risk of major bleedings among patients using DOACs, whereas a higher risk has been reported when pharmacodynamic interacting drugs (i.e., antiplatelet drugs or selective serotonin reuptake inhibitors) are coadministered (Zhang et al., 2020). Significant pharmacokinetic interactions with DOACs have been recently reviewed (Herink et al., 2019; Foerster et al., 2020).

### General Safety and Tolerability

Besides the overall favorable risk–benefit profile of DOACs compared with VKAs across different patients, the risk of gastrointestinal bleedings has been reported higher with dabigatran, rivaroxaban, and high-dose edoxaban; lower with low-dose edoxaban; and unchanged with apixaban (Ruff et al., 2014, 2015; Saraf et al., 2014). Therefore, despite the overall favorable safety profile, bleeding risk remains a critical issue in the selection of the appropriate oral anticoagulant drug. Indeed, there are no head-to-head studies comparing safety and efficacy of different DOACs, and bearing in mind the differences in clinical trial designs as well as patient characteristics enrolled, definitive conclusions cannot be stated due to numerous biases (Lavalle et al., 2020).

Data from postmarketing registries seem to confirm low rates of stroke and major bleeding in “real-world” patients receiving rivaroxaban or edoxaban in routine clinical practice (Camm et al., 2016; de Groot et al., 2020). However, spontaneous treatment emergent adverse events (TEAEs) report databases are showing emerging safety issues. Concerns about nonbleeding TEAEs (e.g., renal, hepatic and severe skin TEAEs, and gastrointestinal symptoms) associated with DOAC therapy have been raised. A sequence symmetry analysis using nationwide data from the French National Healthcare databases has shown, besides the well-known and specific increased bleeding risk, a severe drug-induced liver injury (especially rivaroxaban) and nonbleeding gastrointestinal disorders associated with DOAC treatments (Maura et al., 2018). In contrast, rivaroxaban has demonstrated the best safety profile (lowest ratio between the serious TEAEs rate and the utilization rate of the active principle) in a data analysis from the Italian National Pharmacovigilance Network (Lavalle et al., 2020). In brief, in the whole cohort of 959,231 patients treated with DOACs for several indications, 7,273 have experienced a TEAE, in particular dabigatran 3,342/249,976; rivaroxaban 2,032/317,359; apixaban 1,492/294,721; and edoxaban 407/97,175 (data limited to the biennium 2017–18 for edoxaban). The most common TEAE reported was gastrointestinal disorders (41.2%); to note, 57% were related to gastrointestinal hemorrhages, 8.30% upper abdominal pain, 7.3% rectal hemorrhages, and 5.1% dyspepsia (Lavalle et al., 2020).

## Bridging in Procedural Management and Reversal Agents

Available perioperative data are principally generated from RCT substudies and considering the DOAC pharmacokinetics parameters (short time to peak effect and short half-life), periprocedural bridging is usually not required (Dubois et al., 2017). However, decisions in elective situations about stopping DOACs perioperatively should be based on the thromboembolic risk of the patient as well as general characteristics, the bleeding risk of the invasive procedures, and the DOAC pharmacokinetics profile. Furthermore, coagulation monitoring might be useful in certain populations, considering the elevated interindividual variability of DOAC plasma concentrations (Doherty et al., 2017). The periprocedural management of DOACs has been extensively reviewed in Barnes and Mouland (2018).

Finally, the results of the PAUSE (Perioperative Anticoagulant Use for Surgery Evaluation) study have been published, reporting a functional perioperative management strategy (without heparin bridging or coagulation function testing) related to low rates of major bleeding and arterial thromboembolism (Douketis et al., 2019). Similar results have been shown with a periprocedural management of edoxaban at the discretion of the investigator in the EMIT-AF/VTE study (Colonna et al., 2020).

Reversal agents of DOACs should be considered in urgent surgery, in interventions with high risk of bleeding, and in life-threatening situations (e.g., intracranial hemorrhage). In 2015, the Food and Drug Administration (FDA) has approved idarucizumab, the first-in-class specific reversal agent of DOACs. This monoclonal antibody binds dabigatran specifically with an immediate and complete interruption of the anticoagulant effect (Pollack et al., 2015). Recently, andexanet-α, a recombinant and inactivated coagulation factor Xa, was approved by the FDA for the inhibition of rivaroxaban and apixaban (Heo, 2018).

To date, several specific reversal agents are in preclinical and clinical development; the most advanced candidate is aripazine, a synthetic small molecule with broad activity against both conventional (heparin and low–molecular weight heparin) as well as DOACs (dabigatran, rivaroxaban, apixaban, and edoxaban) (Ansell et al., 2014). Finally, hemodialysis represents a possible nonpharmacological approach to reduce plasma concentration of DOACs characterized by renal elimination and reduced protein binding (i.e., dabigatran) (Enriquez et al., 2016).

## Direct Oral Anticoagulants in Special Populations

### Direct Oral Anticoagulants in Pregnancy

VTE risk increased at least five-fold during pregnancy and the first 6 weeks after childbirth (Unger et al., 2018). For pregnant women in whom VTE prophylaxis is indicated (i.e., women with a history of VTE or with multiple risk factors, such as thrombophilia), low–molecular weight heparin (LMWH) represents the first choice, as well as for acute VTE treatment (Bates et al., 2018; Regitz-Zagrosek et al., 2018). Although theoretically DOACs could be a useful alternative to LMWH, limited data do not support their use during pregnancy and breast-feeding (Bates et al., 2018; Regitz-Zagrosek et al., 2018).

Indeed, clinical risk of DOAC-associated embryopathy is currently unknown and uncertain. Animal and human placenta models suggest a partial transplacental transfer for dabigatran, apixaban, and rivaroxaban, which may raise concerns for reproductive toxicity and indirect effects on fetal blood coagulation (Bapat et al., 2014, Bapat et al., 2015, Bapat et al., 2016). Preclinical studies (described in the summary of product characteristics [SmPCs]) have shown toxic effects of rivaroxaban (including postimplantation pregnancy loss, increased malformations, and placental changes at “clinically relevant plasma concentrations”), dabigatran (preimplantation loss, low fetal body weight, and viability at 5–10x maximum recommended human dose), and edoxaban (gall bladder variations and postimplantation loss at 65x dose) during pregnancy.

Data on DOAC safety in pregnancy and potential teratogenic effects are mostly provided by observational studies, case series, and case safety reports (Beyer-Westendorf et al., 2016). Embryopathy risk has been assessed collecting cases of DOAC exposure in pregnancy from physicians, literature, and pharmacovigilance systems of drug authorities and manufacturers. For 137/233 identified cases in whom rivaroxaban was the most reported DOAC, data outcomes were available. Sixty-seven live births, 31 miscarriages, and 39 elective pregnancy terminations have been observed. Congenital abnormalities were reported in 3/67, 3/31, and 1/39 cases, respectively, and were interpreted as potentially drug-related embryopathy in three cases (Beyer-Westendorf et al., 2016). Potential DOAC teratogenic effects were found by Lameijer et al. (2018), who reported a 4% rate of congenital anomalies associated with rivaroxaban. However, for almost half of the cases describing DOAC-induced teratogenic effects, spontaneous or induced abortion, at least one alternative cause, could potentially explain the occurrence of this serious adverse event (Sessa et al., 2019). Indeed, removing confounding factors from the disproportionality analyses of safety reports registered in *VigiBase*, no statistically significant evidence suggested an increased probability of reporting spontaneous or induced abortion, rather than other adverse events for rivaroxaban. In contrast, this probability seems to be increased for apixaban when compared to warfarin or rivaroxaban (Sessa et al., 2019).

### Direct Oral Anticoagulants Use in Hepatic and Renal Impairments

Patients with renal (i.e., chronic kidney disease [CKD]) and liver impairment have an increased risk of thrombotic events and bleeding complications, and phase III trials have excluded patients with a creatinine clearance (CrCl) less than 25 ml/min or elevated Child–Pugh scores (Chen et al., 2020). Overall, DOACs are eliminated by the kidneys in different amounts, and alterations in hepatic functions may deeply impact on their metabolism (CYP enzymes) and elimination.

As reported above, dabigatran is the most renally eliminated (80%), followed by edoxaban (50%), rivaroxaban (35%), and apixaban (27%) (Steffel et al., 2018). Renal function evaluation and monitoring need to be performed before and during a treatment with DOACs, and dosage corrections should be performed accordingly, preventing also subtherapeutic dosages (Yao et al., 2017). Indeed, inappropriate DOAC dose, due to renal impairment, can increase the risk of bleeding or thrombosis, as reported in up to the 32% of patients (Sanghai et al., 2020).

Considering the lower rate of renal elimination, in patients with only renal impairment (including hemodialysis and end-stage CKD), the FDA does not require dose adjustments for apixaban, except for patients with at least two features among the following: serum creatinine ≥1.5 g/dl, age ≥80 years, or body weight ≤60 kg (Chen et al., 2020). On the other hand, the 2018 European guidelines recommend dose adjustment for apixaban according to CrCl (i.e., CrCL 15–30 ml/min reduced dose and CrCl <15 ml/min avoid treatment) (Steffel et al., 2018).

Dabigatran, edoxaban, and rivaroxaban should go through dose adjustment for renal impairment and ought to be avoided for severe CKD (CrCl <30 ml/min). However, rivaroxaban could be used in patients on dialysis and CKD stage 5 at reduced dose, but several guidelines and consensus documents do not recommend its use (Kumar et al., 2019).

Alterations in hepatic functions impact DOAC metabolism and elimination differently (Qamar et al., 2018). About 75% of apixaban is eliminated through the liver, followed by rivaroxaban (65%), edoxaban (50%), and dabigatran (20%) (Steuber et al., 2019). In patients with severe hepatic disease (based on Child–Pugh scores, grade C), all DOACs are contraindicated, whereas in Child–Pugh grade B, dabigatran, apixaban, and edoxaban could be used with caution. No dose reductions are needed in Child–Pugh grade A for all DOACs (Steffel et al., 2018; Chen et al., 2020). Monitoring coagulation and hepatic functions is recommended before and during treatment.

### Direct Oral Anticoagulants in the Elderly

Elderly patients have a higher risk of both thromboembolic events and anticoagulant-associated bleeding than younger subjects (Lip et al., 2010; Pisters et al., 2010). To date, the available data on DOAC use in elderly have been mainly obtained from prespecified subgroup analyses of larger trials.

Results of the subgroup analyses of RCTs comparing each DOAC with VKAs in patients with VTE or AF confirm the increased risk of thrombotic events and bleeding in the elderly patients, regardless of the anticoagulant strategy used. Dabigatran has shown an increased incidence of bleeding with aging as compared to other agents, although differences in the trial design could explain the difference (Barco et al., 2013). Overall, efficacy and safety results were consistent with those of the main trials, with no significant association between age and treatment (Barco et al., 2013). In some cases, for example, edoxaban, DOACs showed a reduction in major bleeding and in intracranial hemorrhage in patients aged ≥75 years as compared to younger ones (Kato et al., 2016). Recently, in a phase 3, multicenter, randomized, double-blind, placebo-controlled, event-driven trial, a once-daily low dose of edoxaban (15 mg) has been administered in very elderly (≥80 years) patients who were considered inappropriate candidates for a standard oral anticoagulant regimen. In this trial, edoxaban was superior to placebo in preventing stroke or systemic embolism. A higher incidence of major bleeding has been reported with edoxaban (substantially more gastrointestinal bleeding events) compared with placebo, although this difference was not significant (Okumura et al., 2020).

Furthermore, several meta-analyses seem to confirm DOACs’ clinical benefit compared with VKAs also in older patients (Bo and Marchionni, 2020). In a meta-analysis including patients treated for acute VTE and stroke prevention in AF, DOACs showed equal efficacy to VKAs in the subgroup of patients aged ≥75 years. Efficacy data were similar between the elderly subgroup and the total study population. In contrast, different results were reported on bleeding risk. Indeed, apixaban and edoxaban showed a significant reduction in major bleeding risk compared with VKA in both the elderly and total population, whereas similar VKA risks were observed with rivaroxaban. Moreover, both 150 and 110 mg doses of dabigatran in the elderly and the higher dose in the total population were associated with a higher risk of gastrointestinal bleeding than VKA (Sharma et al., 2015).

In a systematic review and meta-regression analysis of older (age >65 years) AF patients on antithrombotic therapy for stroke prevention, 16 studies have compared warfarin with DOACs. These later resulted superior to warfarin in reducing the risk of stroke/TE (HR: 0.81, 95% CI: 0.73–0.89) and mortality (HR: 0.82, 95% CI: 0.74–0.90). Furthermore, DOACs were associated with reduced major bleeding risk (HR: 0.87, 95% CI: 0.77–0.97), although this difference was not significant in real-world studies (RR: 0.82, 95% CI: 0.70–0.95, *n* = 4 in RCTs *vs.* RR: 0.93, 95% CI: 0.75–1.11, *n* = 5 in real-world settings). Major bleeding risk resulted similar to warfarin with dabigatran and rivaroxaban but lower with apixaban and edoxaban (Bai et al., 2018).

Additional real-world data have been obtained by multicenter, prospective register studies such as START2-Register Study, which aims to collect data on effectiveness and safety of any available anticoagulant treatment. The analysis focusing on elderly patients included 272 subjects who were started on anticoagulation (either with VKAs or DOACs) at the age ≥85 years for the occurrence of a VTE episode. During a 429 patient-years follow-up, patients on DOACs had higher rates of bleeding (crude HR 4.7; 95% CI 1.5–15.01) and of thrombotic events (crude HR 4.5; 95% CI 1.5–13.3) than patients on warfarin. However, VTE recurrence was low and similar between the groups, and the mortality rate was markedly reduced in the DOAC cohort (crude HR 0.3; 95% CI 0.1–0.9) (Poli et al., 2019).

### Direct Oral Anticoagulants in Oncologic Patients

Patients with active malignancies are more likely to develop arterial thromboembolism and VTE as well as experiencing bleeding events, due to patient- and cancer-related risk factors (Mosarla et al., 2019). Anticoagulation strategies in cancer-associated VTE are further complicated by potential DDIs between chemotherapy and VKAs, and poor compliance associated with LMWH, the latter being currently the standard treatment. Table 3 summarizes randomized trials comparing VKAs with LMWH (Ay et al., 2019). Evidence on DOAC safety and efficacy in oncologic patients was limited until 2018 to pivotal trial subgroup analyses and subgroup meta-analyses, suggesting similar profiles to those reported in noncancer patients.

**TABLE 3:** Summary of randomized trials comparing VKAs with LMWH (Meyer et al., 2002; Deitcher et al., 2006; Hull et al., 2006; Lee et al., 2015).

Study (time period)	CANTHANOX (3 months)		LITE (12 months)		ONCENOX (7 months)		CLOT (6 months)		CATCH (6 months)	
	VKA	LMWH	VKA	LMWH	VKA	LMVH	VKA	LMWH	VKA	LMWH
Recurrent VTE (%)	4.0	2.8	16.0	7.0	6.5	10.0	17	9	10.5	7.2
Major bleeding (%)	16.0	7.0	7.0	7.0	2.9	9.0	4	6	24	2.7
Mortality (%)	22.7	11.3	47.0	47.0	32.4	32.8	39	41	32.2	34.7
Cancer therapy^a^ (%)	69.3	76.0	NR	NR	32.3^b^ 32.3^c^	56.7^b^ 35.8^c^	76.6	78.7	55.0	50.8
Metastatic disease (%)	52.0	53.5	36.0	47.0	52.9	61.2	68.6	65.9	54.3	55.0
VKA TTR (%)	41		NR		NR		46		47	

VKAs: vitamin K antagonists, LMWH: low molecular weight heparin, TTR: times in therapeutic range, NR: not reported.

a Receiving cancer treatment either at randomization or prior to randomization.

b Percent receiving chemotherapy (trials reports separate percentages for chemotherapy and radiation therapy).

c Percent receiving radiation therapy.

Nowadays, data from larger head-to-head trials comparing DOACs with LMWH in cancer-associated VTE are available. The SELECT-D trial for rivaroxaban, the HOKUSAI VTE-Cancer trial for edoxaban, and the CARAVAGGIO trial for apixaban are briefly described below (Khorana et al., 2017; Raskob et al., 2018; Young et al., 2018).

The multicenter, randomized, and open-label trial “SELECT-D” has enrolled patients with active cancer and symptomatic pulmonary embolism (PE), incidental PE, or symptomatic lower extremity proximal deep vein thrombosis (DVT). Overall, 406 patients have been recruited (58% with a metastatic disease) and randomly assigned to dalteparin or rivaroxaban, with stratification by disease stage. Dalteparin was administered at 200 IU/kg daily during the first month, then 150 IU/kg daily for months 2–6, while rivaroxaban at 15 mg BID for 3 weeks, and then 20 mg QD for a total of 6 months. A total of 216 patients (54%) have completed 6 months of trial treatment, with discontinuation mainly due to death in both arms. Twenty-six patients experienced recurrent VTE (18 treated with LMWH with a 6-month cumulative rate of 11% (range 7–16%) and 4% (range 2–9%) with dalteparin and rivaroxaban, respectively). Regarding safety endpoints, the 6-month cumulative rate of major and clinically relevant nonmajor bleeding was 4% each for dalteparin compared to 6 and 13% for rivaroxaban. Therefore, rivaroxaban seems to reduce the rate of recurrent VTE compared with LMWH in cancer patients, but with a higher risk of bleeding events (Young et al., 2018).

The HOKUSAI VTE-Cancer is a randomized, open-label, non-inferiority trial to evaluate edoxaban *vs.* LMWH for VTE treatment. Overall, 1,050 patients, with acute symptomatic or incidentally detected VTE or PE, were enrolled in 114 centers worldwide; 1,046 were included in the modified intent to treat (mITT) and safety analysis sets.

All patients had a diagnosis of active malignancy within the previous 2 years. The median treatment duration was 211 and 184 days in the edoxaban and dalteparin groups, respectively (*p* = 0.01) (Raskob et al., 2018). Patients were randomized to receive either LMWH for at least 5 days followed by edoxaban (60 or 30 mg QD according to creatinine clearance, body weight, or concomitant P-gp inhibitors) or dalteparin (first month 200 IU/kg QD, then 150 IU/kg QD). A primary outcome event (combination of recurrent VTE and major bleeding at 12 months) occurred in 67/522 patients (12.8%) in the edoxaban group and 71/524 patients (13.5%) in the dalteparin group (*p* = 0.006). Recurrent VTE occurred in 41 (7.9%) and 59 (11.3%) patients in the edoxaban and dalteparin groups, respectively (risk difference 3.4%). Major bleeding occurred in 36 patients (6.9%) in the edoxaban group and in 21 patients (4.0%) in the dalteparin group (risk difference 2.9%) (Raskob et al., 2018).

The CARAVAGGIO trial is a multinational, randomized, controlled, open-label, non-inferiority trial, evaluating apixaban *vs.* dalteparin for the prevention of recurrent VTE in oncologic outpatients. Overall, 1,170 patients with symptomatic or incidentally detected proximal lower limb DVT or PE were enrolled; 1,155 were included in the mITT analysis. Patients were randomly assigned to apixaban or dalteparin monotherapy for 6 months and were stratified according to the type of VTE and timing of cancer diagnosis (active or within the previous 2 years). Apixaban was administered orally at 10 mg BID for the first week, followed by 5 mg BID, while dalteparin subcutaneously at 200 IU/Kg QD for the first month, and then 150 IU/Kg QD (up to 18,000 IU and according to the platelet count). Recurrent VTE occurred in 32/576 patients (5.6%) in the apixaban group and in 46/579 patients (7.9%) in the dalteparin group (*p* < 0.001 for non-inferiority). Major bleeding occurred in 22 patients (3.8%) in the apixaban group (with no fatal episodes) and in 23 patients (4.0%) in the dalteparin group (*p* = 0.60), with 2 fatal episodes. Therefore, apixaban has resulted non-inferior to dalteparin for preventing VTE without increasing the risk of major bleeding in patients with cancer (Agnelli et al., 2020).

A systematic review and meta-analysis assessed DOAC efficacy and safety in the secondary prevention of cancer-associated VTE through data from six RCTs and three prospective cohort studies (Wang et al., 2019). A total of 5,549 patients were included (2,697 treated with DOACs and 2,852 treated with VKAs or LMWH [dalteparin, or enoxaparin]). VTE recurrence occurred in 126/2,697 patients (4.7%) in the DOACs group and in 224/2,852 patients (7.9%) in the traditional anticoagulants group (RR: 0.60, 95% CI: 0.49–0.75, *p* < 0.00001). Bleeding events (major bleeding or clinically relevant nonmajor bleeding) were evaluated in eight of the nine studies. They occurred in 322/2,592 patients (12.4%) treated with DOACs and in 323/2,585 patients (12.5%) treated with VKAs or LMWH (RR: 0.95, CI: 0.67–1.36, *p* = 0.79). These results suggest that DOACs may be more effective than traditional anticoagulants to prevent recurrent VTE in oncologic patients, with no significant difference in safety (Wang et al., 2019).

Therefore, considering evidence from head-to-head RCTs and meta-analyses, DOACs have been added as options for VTE prophylaxis and treatment in cancer patients. Indeed, in the American Society of Clinical Oncology (ASCO) updated guidelines, LMWH, rivaroxaban, and edoxaban are recommended as the first-choice options for VTE treatment. However, due to the observed gastrointestinal bleeding risk, DOACs use should be carefully evaluated in GI cancer patients. Finally, potential DDIs should be checked before starting DOACs, avoiding their administration with chemotherapies which strongly induce or inhibit P-gp or CYP3A4 pathways (Key et al., 2020).

## Discussion

The introduction of DOACs, in the therapy of the primary and secondary prophylaxis of ischemic stroke in patients with NVAF and for the treatment and prevention of DVT/PE, represents an important step in terms of efficacy, safety, and compliance. Previous studies such as ARISTOTELE (Granger et al., 2011), ENGAGE-AF-TIMI-48 (Giugliano et al., 2013), and ROCKET-AF (Patel et al., 2011) demonstrated non-inferiority of apixaban, rivaroxaban, and edoxaban in the prevention of stroke or systemic embolism and in the reduction of major bleeding, especially in the brain, compared to VKAs; furthermore, the RE-LY study (Connolly et al., 2009) conducted with dabigatran 150 mg BID compared to VKA demonstrated a statistically significant reduction in primary endpoint.

One of the main advantages of these drugs is the fixed doses that are established according to renal function and the risk of individual bleeding; moreover, they do not require monitoring of the INR, thus increasing the compliance of frail patients with different functional limitations and multiple comorbidities; in addition, the limited pharmacological interactions of these drugs make them particularly manageable in more complex patients taking several different drugs (Table 4).

**TABLE 4:** Schematic presentation of practical recommendation for clinical DOACs’ use in specific settings.

	Apixaban	Dabigatran	Edoxaban	Rivaroxaban
Elderly	No dose adjustment; coadministration with ASA should be used cautiously	A dose reduction is recommended >75 years	No dose adjustment	No dose adjustment
**Drug–drug interactions**				
P-gp inhibitors (increased risk of bleeding)				
Amiodarone, quinidine, and verapamil	No dose adjustment	Dosing should be reduced	No dose adjustment	Concomitant use with caution
Dronedarone	Concomitant use with caution	Contraindicated	Halved dose	Coadministration should be avoided
Ketoconazole	Not recommended	Contraindicated	Halved dose	Not recommended
Macrolides	No dose adjustment	Dosing should be reduced	Halved dose	Can be clinically significant in high-risk patients
** P-gp inducers (no efficacy)**				
Rifampicin and hypericum	Concomitant use with caution	Concomitant use should be avoided	Concomitant use with caution	Concomitant administration should be avoided
CYP3A4 strong inhibitors or inducers	Not recommended	No DDIs	Not recommended	Not recommended
Miscellaneous				
Antiretroviral protease inhibitors, tyrosine kinase inhibitors, dexamethasone, levetiracetam, and valproic acid	Not indicated	Not indicated	Not indicated	Not indicated (Steffel et al., 2018)
Food–drug interactions	No effect (intake with food discouraged)	Prolongs T_max_ to 2 h (intake with food discouraged)	No effect (intake with food: no official recommendation)	Mean AUC increases to ≈40% (intake with food mandatory)
**Renal impairment**				
CrCL 30–50 ml/min	No dose adjustment	A dose reduction is recommended	Halved dose	No dose adjustment
CrCL 15–30 ml/min	Reduced dose	Avoid treatment	Avoid treatment	Avoid treatment (Steffel et al., 2018)
CrCl <15 ml/min	Not recommended			
**Hepatic impairment**				
Child–Pugh A	No dose reductions are needed	No dose reductions are needed	No dose reductions are needed	No dose reductions are needed
Child–Pugh B	Used with caution	Used with caution	Used with caution	Contraindicated
Child–Pugh C	Contraindicated	Contraindicated	Contraindicated	Contraindicated (Steffel et al., 2018; Chen et al., 2020)
Reversal agents	Andexanet-α	Idarucizumab and hemodialysis		Andexanet-α

AUC: area under the curve, ASA: acetylsalicylic acid, CrCl: creatinine clearance, DDIs: drug–drug interactions, P-gp: P-glycoprotein.

If not different specified, data reported can be found in SmPC.

The DOAC most affected by DDIs is dabigatran, due to the role of P-gp in its absorption, and for drugs that increase its absorption that are often used for the treatment of NVAF such as amiodarone and verapamil (Gong and Kim, 2013; Bernier et al., 2019; Corsini et al., 2020), rivaroxaban and apixaban are also substrates of BRCP transporters (Fawzy and Lip, 2019). Instead, edoxaban can be administered simultaneously with drugs used to treat NVAF (Corsini et al., 2020). Dabigatran and rivaroxaban represent the DOACs most affected by the interaction with food compared to apixaban and edoxaban (Kubitza et al., 2006; Gnoth et al., 2011; Stampfuss et al., 2013).

In the case of perioperative or intracranial major bleeding during treatment with DOACs, we have several specific reversal agents: idarucizumab for dabigatran (Pollack et al., 2015) and andexanet-alfa for edoxaban, apixaban, and rivaroxaban (Heo, 2018), and if these agents are not sufficient, hemodialysis could represent a useful therapeutic option to eliminate from the circulation those drugs with a high rate of renal elimination and low plasma protein binding such as dabigatran (Enriquez et al., 2016).

The efficacy of DOACs in preventing cardiovascular events has also been tested in high-risk populations such as patients with PAD undergoing revascularization. In fact, in the VOYAGER-PAD study, patients treated with rivaroxaban compared to patients treated with VKA obtained a 15% reduction in cardiovascular events such as acute limb ischemia, amputation due to vascular causes, myocardial infarction, ischemic stroke, or cardiovascular death but with an increase in major bleeding (Bonaca et al., 2020).

Historically, there is no indication for the use of DOACs in patients with mechanical heart valves; however, several subgroup analyses from RCTs and cohort studies do not preclude their use in patients with bioprosthetic valves (Steffel et al., 2018). Recently, the RIVER study, conducted in patients with AF and bioprosthetic mitral valve, demonstrated the non-inferiority of rivaroxaban 20 mg QD compared to dose-adjusted warfarin (target INR, 2.0–3.0) on the primary outcome of death, major cardiovascular events (stroke, transient ischemic attack, systemic embolism, valve thrombosis, or hospitalization for heart failure), or bleeding over 12 months (Guimarães et al., 2020). An important indication is coming for the use of these drugs in a category of postsurgical patients otherwise forced to take VKAs.

In addition, a population often not considered in pivotal studies is represented by the elderly; however, age and AF represent the main independent risk factors for stroke (Wolf et al., 1991; Go et al., 2001). Although there are indications for the use of DOACs in elderly patients and numerous meta-analyses have confirmed their superiority over VKAs in elderly patients, their management is difficult due to comorbidities, polytherapy, the risk of falls, and bleeding (Bo and Marchionni, 2020). Recently, a phase 3 study conducted in patients older than 80 years, deemed not eligible for anticoagulation, has compared edoxaban 15 mg/day *vs.* VKAs. A statistically significant reduction in strokes was observed without a significant increase in major bleedings (Okumura et al., 2020). The results of this study are particularly encouraging and provide us with useful information for the safe and effective management of a category of patients too often forgotten in large randomized clinical trials, but well-represented in real clinical settings.

Pregnant women represent a high-risk population for thromboembolic events; the first therapeutic choice for prophylaxis and treatment of DVT is represented by LMWH. The data available on the safety of DOACs in pregnancy and potential teratogenic effects come from case series or case safety reports (Beyer-Westendorf et al., 2016); the data so far available are not sufficient to provide clinical recommendations on the use of DOACs in pregnancy; future observational or database studies will clarify this aspect.

Patients with active cancer represent a high-risk population for thromboembolic events and bleeding due to both disease and individual risk factors. Because of the numerous DDIs, the recurrence rates of DVT/PE or bleeding, the intestinal absorption, and the management difficulties, VKAs are not the first short- and long-term therapeutic choices in patients with cancer-associated DVT/PE. These limits are overcome by LMWH which has similar or lower rates of DVT/PE recurrence and bleeding than VKAs in patients with cancer-associated DVT/PE; its efficacy is unaffected by gastrointestinal absorption, and it has minimal interaction with chemotherapeutic agents. Therefore, LMWH represents the first therapeutic choice for the short- and long-term management of cancer-associated VTE but is limited by poor patient compliance. The SELECT-D study for rivaroxaban, the HOKUSAI VTE-Cancer study for edoxaban, and the CARAVAGGIO study for apixaban compare DOACs with LMWH in cancer-associated VTE (Khorana et al., 2017; Raskob et al., 2018; Young et al., 2018). In all these studies, there was a reduction in cancer-associated DVT/PE relapses in the groups treated with DOACs compared to the group treated with VKAs, but at the same time, there was an increase in major bleeding. The results of these studies were confirmed by a recent meta-analysis which however found no statistically significant differences in major bleeding (Wang et al., 2019). In light of the data illustrated above, the ASCO and its guidelines recommend the use of LMWH, edoxaban, and rivaroxaban as the first therapeutic choice in the treatment of DVT/PE, with particular caution in patients with gastrointestinal cancer due to the risk of bleeding that is strongly associated with cancer localization (Key et al., 2020).

In the next few years, we could obtain indications for their use in peripheral arterial disease (PAD) or in patients with coronary artery disease (CAD), given the results of the VOYAGER-PAD study (Bonaca et al., 2020); indeed, there are many patients who, despite optimal medical therapies, undergo numerous and repeated cardiovascular events; thus, the use of DOACs could represent an added value in terms of effectiveness and safety.

It is plausible that one of the future research fields will consist in the use of this pharmacological class in patients with severe renal and hepatic insufficiency as they represent a population at high risk of thromboembolic and hemorrhagic complications. As previously mentioned, a population with a high thromboembolic and simultaneously hemorrhagic risk is represented by the very elderly who could benefit from a reduced dosage of DOACs as already demonstrated by the ELDERCARE-AF study with edoxaban 15 mg QD *vs.* placebo, thus obtaining a lower rate of stroke and major bleeding and a significant improvement in the quality of life. Additionally, another issue deserving a more thorough investigation is how gender-related differences in pharmacokinetic, pharmacodynamic, and clinical features may affect drug response and the incidence of adverse events. To date, limited data on DOACs exist in this field. Indeed, subgroup analyses of larger trials might be underpowered to state conclusions, also due to women underrepresentation in cardiovascular clinical trials. Recently, in a meta-analysis of RCTs comparing DOACs with standard antithrombotic therapy for NVAF treatment, DOACs were associated with a significantly lower risk of major bleeding in women than men (RR = 0.86, 95% CI = 0.78–0.94) (Raccah et al., 2018). A lower risk of intracranial hemorrhages in women treated with DOACs has been also observed in a population-based cohort study (Law et al., 2018). Moreover, indirect comparisons showed significant differences among specific DOACs between women and men, suggesting the relevance of dose adjustment according to body weight (Raccah et al., 2018). Finally, as DOACs differ significantly in pharmacokinetic and safety profiles, the importance of head-to-head trials, still lacking in the literature, to directly compare their safety and efficacy should be highlighted. Direct comparison studies are mandatory to better define the specific clinical profile of each DOAC and address the right patient-tailored therapy.

## Glossary

ACS: acute coronary syndrome
AF: atrial fibrillation
ASCO: American Society of Clinical Oncology
BCRP: breast cancer–resistant gene protein
BID: bis in die (twice daily)
CAD: coronary artery disease
CI: confidence interval
CKD: chronic kidney disease
CrCl: creatinine clearance
CYP: cytochrome P450
DDIs: drug–drug interactions
DOACs: direct oral anticoagulants
DVT: deep vein thrombosis
FDA: Food and Drug Administration
GFR: glomerular fraction rate
GI: gastrointestinal
HR: hazard ratio
INR: international normalized ratio
ISTH: International Society on Thrombosis and Haemostasis
LMWH: low–molecular weight heparin
mITT: modified intent to treat
NVAF: nonvalvular atrial fibrillation
OR: odds ratio
PAD: peripheral arterial disease
PCI: percutaneous coronary intervention
PE: pulmonary embolism
P-gp: permeability glycoprotein
QD: quaque die (once daily)
RCTs: randomized clinical trials
RR: relative risk
SmPC: summary of product characteristics
SULT: sulfotransferase
TE: thromboembolism
TEAEs: treatment emergent adverse events
TIMI: thrombolysis in myocardial infarction
V_d_: volume of distribution
VKAs: vitamin K antagonists
VTE: venous thromboembolism
